# Delineation and Modulation of the Natural Killer Cell Transcriptome in Rhesus Macaques During ZIKV and SIV Infections

**DOI:** 10.3389/fcimb.2020.00194

**Published:** 2020-04-29

**Authors:** Malika Aid, Daniel R. Ram, Steven E. Bosinger, Dan H. Barouch, R. Keith Reeves

**Affiliations:** ^1^Center for Virology and Vaccine Research, Beth Israel Deaconess Medical Center, Harvard Medical School, Boston, MA, United States; ^2^Emory Vaccine Center, Yerkes National Primate Research Center, Atlanta, GA, United States; ^3^Ragon Institute of Massachusetts General Hospital, MIT, and Harvard, Cambridge, MA, United States

**Keywords:** NK cells, rhesus macaques, RNA-seq, SIV, ZIKV

## Abstract

Natural killer (NK) cells are crucial regulators of antiviral and anti-tumor immune responses. Although in humans some NK cell transcriptional programs are relatively well-established, NK cell transcriptional networks in non-human primates (NHP) remain poorly delineated. Here we performed RNA-Seq experiments using purified NK cells from experimentally naïve rhesus macaques, providing the first transcriptional characterization of pure NK cells in any NHP species. This novel NK cell transcriptomic signature (NK RMtsig) overlaps with published human NK signatures, allowing us to identify new key signaling and transcription factor networks underlying NK cell function. Finally, we show that applying NK RMtsig to an unrelated rhesus macaque cohort infected with SIVmac251 or ZIKV can sensitively detect NK cell repertoire perturbations, thus confirming applicability of this approach. In sum, we propose this NHP NK cell signature will serve as a useful resource for future studies involving infection, disease or treatment modalities in NHP.

## Introduction

Natural killer (NK) cells are some of the earliest responders to viral infections and form an essential component of the anti-viral innate immune response (Alter and Altfeld, 2009; Gandhi et al., 2010). Though they play a substantial role in the regulation of viral infections and nascent neoplasms, these immune cells remain poorly investigated compared to their adaptive counterparts, T cells and B cells. Although NK cells are considered a heterogenous population, they can be broadly classified as cytokine-producing or cytotoxic cells (Manickam et al., 2019). Their functional and phenotypic characterization may also vary depending on tissue localization or disease state allowing NK cells to be versatile in their responses. In many non-human primates (NHP), NK cells are defined as being CD14^−^CD20^−^CD3^−^CD159A/C^+^ and in rhesus macaques the predominant population in blood can be additionally defined by the expression of CD16, whereas in certain tissues CD56 is generally upregulated on the cell surface of NK cells (Reeves et al., 2010; Manickam et al., 2019).

In humans and NHP, NK cells have been shown to play a critical role in controlling HIV and SIV infections mainly by direct targeting of virally-infected cells through activating receptors, or via antibody dependent cell-mediated cytotoxicity (ADCC) (Shieh et al., 2001; Schafer et al., 2015). In SIV-infected monkeys, activated NK cells with cytolytic functions in peripheral blood correlated with better control of infection (Shieh et al., 2001). During the course of HIV/SIV infection it has been observed that NK cells become dysfunctional, especially during the chronic stages of infection (Alter and Altfeld, 2009; Brunetta et al., 2009; Schafer et al., 2015). The exact mechanisms of how HIV/SIV infection alters NK cell function are still unclear, though evidence suggests this may be a result of broader effects from altered CD4+ T cell levels (Luo et al., 2017). Interestingly, NK cells have also been shown to be capable of eliciting memory recall responses in the context of SIV infection in rhesus macaques (Reeves et al., 2015) and to CMV infection in humans (Foley et al., 2012; Hammer et al., 2018). This may provide an opportunity for developing novel vaccine strategies and as a result it is important to understand the mechanisms regulating NK cell function, especially in the context of viral infection.

To date a small number of studies have been performed in humans to define the transcriptional profile of human NK cells using microarrays or RNA-Seq experiments (Nakaya et al., 2011; Li et al., 2014; Newman et al., 2015; Costanzo et al., 2018; Crinier et al., 2018) whereas in NHP most of the NK cell transcriptional characterization has been carried out using Fluidigm or RT-PCR (Hong et al., 2013). The NK cell transcriptome has been characterized more extensively in mice (Zhou et al., 2017; Crinier et al., 2018), but since the differences between mouse and rhesus are greater than those between rhesus and human we focused on comparing our data with several human datasets for this work. NK cell gene signatures identified in these human studies are currently used as a reference to study NK cells and to screen for the enrichment of NK cell markers in both human and NHP studies. Since NHP are largely used as a model to study human biology and infectious diseases, particularly HIV, it is crucial to define an NHP NK cell signature and to understand how NK cell transcriptional responses in NHP may also change during infection. Therefore, we performed RNA-Seq experiments to define the NK cell transcriptomic identity in NHP and provide a resource for NHP research. To our knowledge, this is the first large scale study to define the transcriptomic profile of NK cells in a rhesus macaque model.

## Materials and Methods

### Ethics Statement

This manuscript utilized banked animal PBMC samples and no new animals were acquired specifically for the analyses described herein. All original animals were housed at the New England Primate Research Center of Harvard Medical School in accordance with the rules and regulations of the Committee on the Care and Use of Laboratory Animal Resources. Animals were fed standard monkey chow diet supplemented daily with fruit and vegetables and water ad libitum. Social enrichment was delivered and overseen by veterinary staff and overall animal health was monitored daily. All studies reported here were performed under protocol #04637 which was reviewed and approved by the Harvard University IACUC. When necessary, macaques were immobilized with ketamine HCl (Parke-Davis) at approximately 10 mg/kg and injected intramuscularly after overnight fasting. Blood samples were collected using venipuncture.

### Animals

Experimentally naive male age-matched Indian-origin rhesus macaques were analyzed in this study. All animals were colony-housed at the New England Primate Research Center and were free of simian retrovirus type D and simian T-lymphotropic virus type 1. Blood samples were collected in EDTA-treated tubes, and peripheral mononuclear cells (PBMCs) were isolated using standard density gradient centrifugation over lymphocyte separation media (MP Biomedicals, Solon, OH) and any contaminating red blood cells were lysed using hypotonic ammonium chloride solution. PBMCs were frozen in 90%FBS + 10% DMSO (Sigma) in the LN_2_ vapor phase.

### Antibodies and NK Cell Sorting

All antibodies were purchased from BD Biosciences unless otherwise specified. For NK cell sorting the following cell antigens were used: CD14 (MøP9), CD20 (L27), CD3 (SP34.2), CD159a (Z199, Beckman Coulter). Additionally, the AQUA viability assay was used to identify viable cells. PBMC were thawed and stained with AQUA viability stain, followed by sorting antibodies as detailed above. NK cells were then sorted into cold R10 using the following gating strategy in order to identify NK cells: CD14^−^CD20^−^CD3^−^CD159A/C^+^ (Reeves et al., 2010; Supplementary Figure 1).

### RNA Isolation

The sorted NK cells were pelleted (500 × g, for 10 min) and lysed by vortexing for 1 min in cold supplemented RLT buffer (RLT + β-MeOH) using the following ratio: 50 μL cells in R10 and 350 μL RLT buffer, 4°C. Samples were then immediately frozen at −80°C. RNA was extracted from these samples using the RNeasy Micro kit (Qiagen) with on-column DNase digestion. RNA quality was assessed using an Agilent Bioanalyzer.

### Library Preparation and RNA-Seq Processing

Five (5) nanograms of total RNA was used as input for cDNA synthesis using the Clontech SMART-Seq v4 Ultra Low Input RNA kit (Takara Bio) according to the manufacturer's instructions. Amplified cDNA was fragmented and appended with dual-indexed bar codes using the NexteraXT DNA Library Preparation kit (Illumina). Libraries were validated by capillary electrophoresis on an Agilent 4200 TapeStation, pooled at equimolar concentrations, and sequenced on an Illumina HiSeq3000 at 100SR, yielding 20–25 million reads per sample. Alignment was performed using STAR algorithm version 2.5.2b (Dobin et al., 2013). Transcripts were annotated using MacaM assembly and annotation of the Indian rhesus macaque genome (Zimin et al., 2014) (http://www.unmc.edu/rhesusgenechip/index.htm#NewRhesusGenome). Transcript abundance estimates were calculated internal to the STAR aligner using the algorithm of htseq-count generating the raw read count table (Anders et al., 2015).

### Gene Desirability Score Function

To define our NK cell signature from rhesus macaques, first we converted the raw gene expression matrix into a count per million (cpm) expression matrix in order to normalize for library size differences between samples. Then, we filtered all genes with a cpm count <1 across all animals to ensure each gene is expressed at some minimal level across all animals. This step generated an initial signature of approximately 9,000 genes. Next, we applied the approach implemented in the desiR package (https://cran.r-project.org/web/packages/desirability/desirability.pdf) (Derringer and Suich, 1980) to select for the top NK cell expressed genes. This approach ranks genes using their overall importance across all samples and assigns a desirability score between 0 and 1 to each gene.

For each gene, we defined its average cpm count as *R*_*i*_*(x)*. A desirability function *D*_*i*_*(R*_*i*_*)* assigns a score between 0 and 1 to all possible values of *R*_*i*_, with:

*D*_*i*_*(R*_*i*_*)* = 0 representing the lowest undesirable value of *R*_*i*_ and

*D*_*i*_*(R*_*i*_*)* = 1 representing the highest desirable value of *R*_*i*_.

The desirability function *D*_*i*_*(R*_*i*_*)* varies depending on whether a particular response *R*_*i*_ is to be maximized, minimized or equal to a specific threshold.

Let *L, H* and *T* be the lower, upper, and target values, respectively, that are desired for a response *R*_*i*_, with *L* ≤ *T* ≤ *H*, where *L, H* and *T* represent threshold values defined by the user. We implemented a desirability function that maximizes the score assigned to important genes (genes with high average cpm count) and defined the desirability function for each gene as:

(1) $$\begin{array}{l} Di\left(Ri\left(x\right)\right)=\left\{ \begin{array}{c} 0\;if\;Ri\left(x\right)<L\;\\ \frac{\left(Ri\left(x\right)-L\right)}{\left(T-L\right)}\;if\;L\;\\ 1.0\;if\;Ri\left(x\right)\geq T \end{array}\;\leq \;Ri\left(x\right)\;\leq T\right. \end{array}$$

Where, *Di(Ri(x))* is the desirability score for gene *x*_*i*_.

In order to select *L* and *T* parameters, first we plotted the histogram of average cpm count distribution of all genes in our initial signature (9,000 genes) and selected the minimum cut-off *L* equal to 1 and the maximum cut-off *T* equal to 6 (Supplementary Figure 2). Although the choice of these two parameters may seem random, we selected the values of *L* and *T* based on the specific distribution of our data by (1) filtering more genes with low cpm count and (2) setting up a a maximal value *T* that reflects the inflection point starting from which a gene is considered to be highly significant and assign a score of 1 to all the genes with an average cpm count higher than this maximal threshold. Also, because we did not prioritize only genes with maximal desirability score (*DesiR* = *1*) we think these parameters represent an acceptable trade-off to distinguish between high and low desirable genes as shown in Supplementary Figure 2. Next, We used the DesiR package and its *d.high* function to assign a score to all genes in our initial signature. This function generated desirability scores ranging from 1 (highly desirable gene) to 0 (not desirable gene). We selected the top genes (5,627 genes) with a desirability score of 0.70 or higher as the final NK cell signature designated by the NK cell rhesus macaque transcriptomic signature NK RMtsig (Supplementary Table 1). Although, we used these highly desirable genes for all the analyses conducted in this study, we think that the remaining genes (desirability score <0.70) are also important and need to be considred when screening for the enrichment of NK cell signatures (Supplementary Table 2).

### Pathways Enrichment Analyses

We used the overlapping test implemented in the GeneOverlap R package (https://github.com/shenlab-sinai/geneoverlap) to assess the overlap of our NHP NK cell signature with published collections of gene sets and pathways (Chaussabel et al., 2008; Liberzon et al., 2011; Nakaya et al., 2011; Newman et al., 2015; Costanzo et al., 2018; Yang et al., 2019). All gene sets and pathways that were enriched with a false discovery (FDR) q value cut-off of 0.05 were selected and the overlapping genes between these significant signatures and our NK RMtsig were used to generate heatmaps and gene networks.

### Gene Network Analyses

All gene networks were generated using the DyNet Analyzer tool implemented under Cytoscape version 3.6.0 (https://cytoscape.org). For gene annotation, we used GeneMANIA version 3.3.1 (http://genemania.org), Genecards (https://www.genecards.org), Reactome database and CluGo tool implemented under Cytoscape version 3.6.0. For transcription factors (TFs) enrichment analyses, we used the database pscan (http://www.fiserlab.org/tf2dna_db/) and selected TF targets from humans and NHP studies only.

### Statistical Analysis

All the analyses in this paper were generated using the following R packages: limma, corrplot, DESeq2, heatmap.2, pheatmap, circlize, and GeneOverlap available via the Bioconductor web site at https://www.bioconductor.org. RNA-Seq analysis was performed using DESeq2 R package (Love et al., 2014). Correlation plots were generates using the R package corrplot with the following parameters (method=pie, correlation = Spearman, significance *p* value level sig.level = 0.05 and interval confidence conf.level = 0.95). Microarray data from previously published independent studies of SIV-infected rhesus macaques in blood, LN and FRT and from ZIKV infected rhesus macaques in blood were analyzed using the limma R package as described previously (Barouch et al., 2016; Aid et al., 2017). First, differential gene expression analysis was performed at days 1, 3, 7, and 10 following SIV infection compared to day 0 in blood, LN and FRT tissues and at days 2, 4, 6, and 14 following ZIKV infection in blood. Next, we overlapped our NK RMtsig with genes modulated by SIV or ZIKV and those NK RMtsig Genes that were significantly increased or decreased (Benjamini–Hochberg adjusted *p* < 0.05) following infection.

## Results

### Transcriptomic Profiling of NK Cells in Rhesus Macaques by RNA-Seq

To provide an unbiased transcriptomic profile of purified NK cells, we performed RNA-Seq experiments using sorted peripheral NK cells from experimentally naive rhesus macaques. NK cells from Indian-origin rhesus macaques were sorted by the CD14^−^CD20^−^CD3^−^CD159A/C^+^ cell phenotype as shown in Supplementary Figure 1 as previously defined by our group (Reeves et al., 2010). From our analyses we selected the top genes with a desirability score higher than 0.70 to define our NHP NK cell signature (Supplementary Table 1). Genes with a desirability score <0.70 are shown in Supplementary Table 2. In the remaining sections, we will refer to this as NK cell rhesus macaques transcriptomic signature (NK RMtsig).

**Figure 1:**
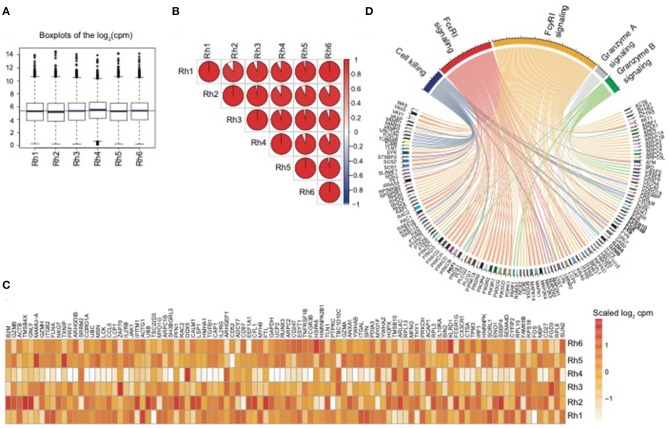
NK RMtsig expression profile is consistent across all animals and shows enrichment of published NK cell markers and signaling pathways. **(A)** Boxplots showing the distribution of the log2(cpm counts) for each sample using the NK RMtsig. The blue horizontal line represents the median (cpm counts) across all animals. **(B)** Correlation plot showing the pairwise Spearman correlation of the NK RMtsig expression across all six animals. Pie charts represent the degree of the Spearman correlation ranging from blue (negative correlation) to red (positive correlation), where full pie corresponds to maximal correlation (*r* = 1 and empty pie represents no correlation (*r* = 0). **(C)** Heatmap representing the scaled log_2_ (cpm count) of the top 100 highly expressed gene from the NK RMtsig. **(D)** Circular plot of major NK cell signaling pathways enriched in the NK RMtsig. Pathways were shown in different colors. Genes within each pathway were shown with edges of the same color.

In order to assess the overall expression similarity of the NK RMtsig across all animals, we performed a pairwise Spearman analysis correlating the expression of all genes within the NK RMtsig between each pair of animals. We observed that the expression of the NK RMtsig was highly similar across all animals as shown by the boxplots of the cpm count distribution and the Spearman correlation plot (Figures 1A,B, Supplementary Figure 6). These results suggest little to no difference in the transcriptomic profile, and that the expression of the NK RMtsig was quite uniform with minimal animal-to-animal variation. Next, we tested if our NK RMtsig was enriched in known major NK cell markers. We found that several well-established NK cell genes were among the top genes with high desirability scores including *GZMB, GZMA, NKG7, PRF1, CCL5, KLRD1, KLRC1, KLRC3, KLRF1, KIR2DL4*, and *FCGR3B, IL2RB, EOMES, LAIR1*, and *CD2* (Figure 1C, Supplementary Table 1). These genes have been well characterized to play roles in NK cell effector immune responses as well as in normal NK cellular differentiation (Costanzo et al., 2018). In order to further characterize our NK RMtsig, we tested for the enrichment of known NK cell signaling pathways in the NK RMtsig. This approach allowed us to assess whether NK RMtsig overlaps with published human NK signaling pathways. We used the overlapping test as described in the Methods section and NK cell molecular signatures from the MSigDB C2 gene sets and the NK cell signatures from the blood transcription modules collection (Chaussabel et al., 2008; Liberzon et al., 2011). While it was not surprising to see many pathways enriched in NK RMtsig, we observed significant enrichment of several key, well-characterized NK cell pathways as determined by the overlapping test significance (FDR < 0.05). These significantly enriched pathways included granzyme B and granzyme A signaling (*PRF1GZMB, GZMA, BID, CASP3, CASP8*), cell killing (*MAP2K1, TUSC2, TUBB4B, VAMP2, PRDX1*), FcγRI and FcεRI signaling (*FCER1G, FCGRB, FGR, FYB, PIK3R5, PIK3R4, PIK3R1, PIK3R2, VAV3, VAV1, SOS1, SOS2*) (Figure 1D). Our results showed that NK RMtsig overlaps significantly with published human NK cell markers and with well-established NK cell signaling pathways.

### Genes Expressed in Rhesus Macaque NK Cells Display Significant Overlap With Published Human NK Cell Signatures

**Figure 2:**
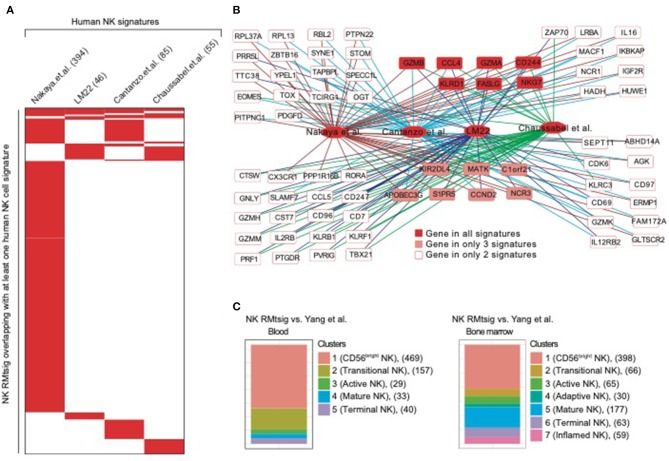
NK cell signature from NHP overlaps with published NK signatures in humans. **(A)** Heatmap showing the overlap between NK RMtsig and published NK signatures from several human studies. Each column represents an NK signature from humans and each row represents a common gene with the NK RMtsig, depicted in red if the gene is present in the human signature or in white if the gene is absent from the human signature. For each human signature, the number of overlapping genes with the NK RMtsig is shown between parentheses. **(B)** Genes Network of NK genes common to both NHP and humans from the signatures shown in panel A. **(C)** Graphical representation of the overlap between the NK RMtsig and NK cell populations signatures derived from human in blood and bone marrow using single cell RNA-Seq experiments.

To further assess the degree of overlap between NK RMtsig and published NK cell signatures derived from *in vivo* and *in vitro* human studies, we conducted a meta-analysis where we compared NK RMtsig with several human NK cell signatures derived from microarrays or RNA-Seq experiments in humans (Figure 2, Supplementary Table 3). First, we compared NK RMtsig with the human NK cell module from the LM22 database (Newman et al., 2015). Among the 79 human NK cell genes in the LM22, 46 genes were found in the NK RMtsig (Figures 2A,B). These genes include major NK cell markers such as *PRF1, NKG7, CCL5, GZMB, KLRD1, KLRC3, KLRB1, KIR2DL4, CD69*, and TBX21. In a second study, Nakaya and colleagues (Nakaya et al., 2011) conducted a meta-analysis using publicly available microarrays datasets of major PBMC populations, including T cells, B cells, monocytes and NK cells. They analyzed two different NK cell microarray datasets. In the first dataset, NK cells from tonsil were obtained from patients undergoing routine tonsillectomy. In the second dataset, primary NK cells (CD56^+^/CD16^+^ and CD3^−^) were isolated from PBMC from seven healthy donors. Out of the 1469 NK genes reported in this microarrays analysis, 394 genes overlapped with NK RMtsig (Figures 2A,B), including several interferon and cytokine signaling genes. In a third study, Costanzo and colleagues (Costanzo et al., 2018) sorted NK cells (CD56^dim/neg^ and CD57^+^NKG2C^+^) from PBMC from six healthy donors under various activating conditions. Then gene expression profiling was performed to define the gene signature of responding NK cells compared to unstimulated NK cells. In this study, the authors reported three NK cell signatures: genes upregulated following IL12 and IL-18 treatment (IL12_IL18 NK, 79 genes), NK cytolytic genes (98 genes) and genes upregulated during NK coculture with K562 (K562 NK, 67 genes). Among these three signatures 41, 33 and 11 genes overlapped with NK RMtsig, respectively, and all include *CCL4, BCL2, CD244, EOMES, FALSG, IL21R, IL2RG, NFKB1, IL12RB2, IL4R, IL16, TOX and PRDX1* (Figures 2A,B). Finally, we assessed the overlap between NK RMtsig with a module of NK cell genes (78 genes) from the molecular signatures database (Chaussabel et al., 2008). Fifty-five genes out of 78 were found in NK RMtsig and include *CX3CR1, EOMES, NLRC3, TIGIT, TGFBR3, RORA, STAT4* (Figures 2A,B).

A recent study by Yang and colleagues (Yang et al., 2019) using single cell RNA-Seq of NK cells sorted from human bone marrow and blood, identified the transcriptional profiles of several NK cell populations including (i) CD56 bright, (ii) transitional, (iii) active, (iv) mature and (v) terminally differentiated NK cells. Therefore, we also compared NK RMtsig to these different NK cell populations signatures (Figure 2C). In bone marrow, 398 genes in cluster 1 (CD56^bright^ NK), 66 genes in cluster 2 (transitional NK), 65 genes in cluster 3 (active NK), 30 genes in cluster 4 (adaptive NK), 177 genes in cluster 5 (mature NK), 63 genes in cluster 6 (terminal NK) and 59 genes in cluster 7 (inflamed NK) were found in NK RMtsig. Similarly in blood, 469 genes in cluster 1 (CD56^bright^), 157 genes in cluster 2 (transitional), 29 genes in cluster 3 (active), 33 genes in cluster 4 (mature) and 40 genes in cluster 5 (terminally differentiated) were found in NK RMtsig. Together, these analyses showed a significant overlap between NK RMtsig and published human NK cell signatures generated from independent studies, thus confirming the validity of our approach and providing this signature as a sensitive tool to probe both human and NHP data sets.

### NK RMtsig Is Enriched in Pathogen-Associated Molecular Pattern Sensing, Metabolism, Cell Cycle and Survival Pathways

**Figure 3:**
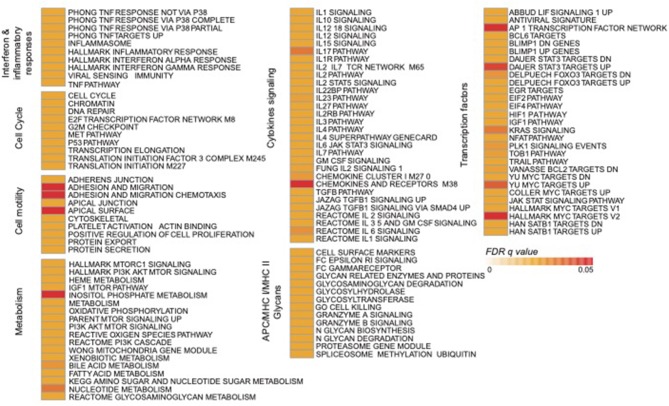
NK RMtsig revealed expression of a broad range of innate and adaptive immune pathways. Diagram showing all the significant pathways (FDR q value of the overlapping test < 0.05) enriched within the NK RMtsig. Pathways were grouped into modules of similar or close molecular function. The color gradient represents the gene overlapping FDR q value, ranging from light orange (FDR = 0) to dark red (FDR = 0.05).

**Figure 4:**
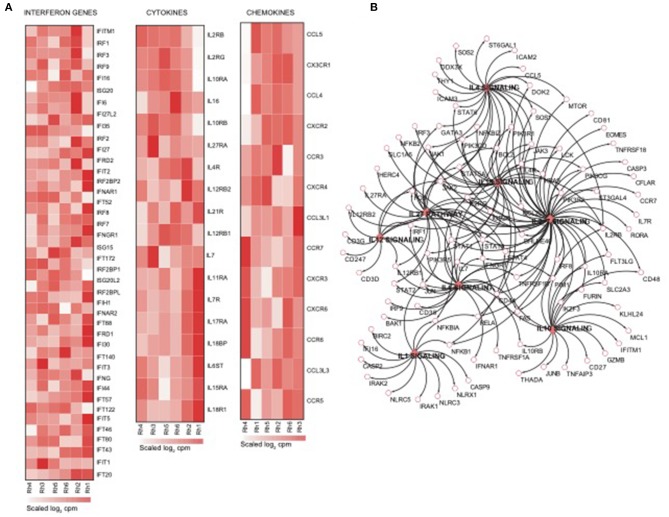
NK cell transcriptomic profile is enriched in interferon genes, cytokines and chemokines and their downstream signaling. **(A)** Heatmaps representing the normalized expression of interferon genes, cytokines and chemokines enriched in the NK RMtsig. Each column represents an individual animal and each row represents a gene. **(B)** Genes network representing the cytokines downstream signaling enriched in NK RMtsig. Genes shared by two pathways or higher were highlighted in full red boxes. Unique genes for each pathway were shown in empty boxes. The red color gradient corresponds to the number of pathways which a specific gene belongs.

**Figure 5:**
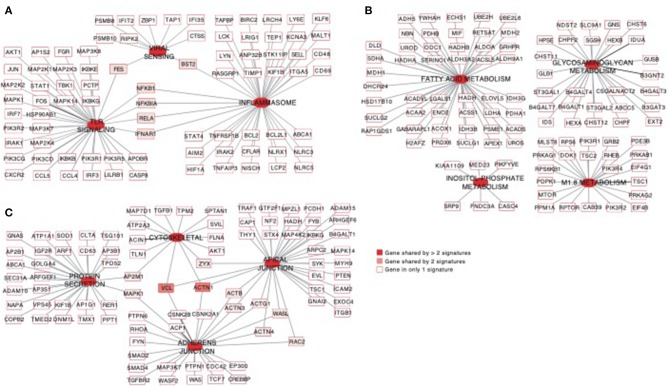
Pathways of viral sensing and TLR signaling, metabolism, cell motility and protein export are enriched in the NK RMtsig. **(A–C)** Gene networks representing pathways of viral sensing, TLR signaling, inflammasome **(A)**, metabolism pathways **(B)** and cell motility and protein export pathways **(C)** enriched in the NK RMtsig. Genes shared by 2 or more pathways were highlighted with full red boxes, where red color gradient represents the number of pathways that share this gene. Unique genes for each pathway were shown in empty boxes.

Using the same overlapping approach as described above, we tested for the enrichment of additional signaling pathways in NK RMtsig. We found that modules of cell cycle, cell motility and export, interferon and inflammatory responses, metabolism, glycan signaling and antigen presentation and processing including MHC I and MHC II pathways were all significantly enriched in NK RMtsig (Figure 3), as were multiple interferon, cytokine and chemokine genes (Figure 4). Moreover, downstream signaling by cytokines involved in the inflammatory response (*IL-1* signaling pathway), immune inhibition (*IL-10, IL-27* and *TGF-*β signaling pathways), cell activation and proliferation (*IL-2* and *IL-7* signaling pathways) and others were also enriched in NK RMtsig (Figure 4A). We also observed that several signaling pathways associated with pathogen recognition and rapid innate immune responses were enriched in NK RMtsig, including TLRs, those shared with other intracellular pathogen-associated molecular pattern sensors as well as the inflammasome (Figure 5A). In addition, we observed enrichment in metabolic pathways including signaling by mechanistic target of rapamycin (*mTOR*) and fatty acid metabolism (Figure 5B) as well as pathways involved in functional NK cell responses, including protein secretion, synapse formation (Figure 5C), and several markers of glycosylation signaling (Supplementary Figure 3).

**Figure 6:**
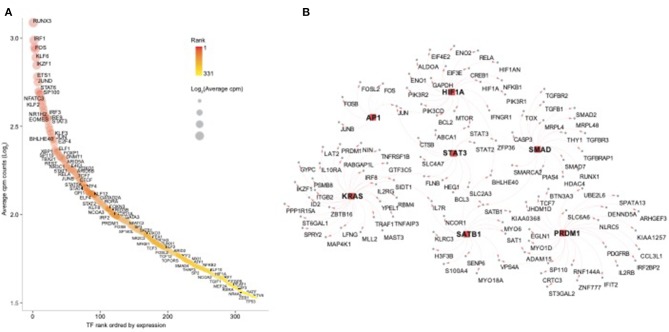
Transcription factors regulating epigenetic modifications, proliferation, cell homeostasis, T follicular helper cell and B cell functions are enriched in the NK RMtsig. **(A)** Scatter plot showing the 332 human transcription factors (TFs) enriched in NK RMtsig. All TFs were ranked using their expression from high to low. X axis represents gene rank and Y axis represents the Log_2_ transformation of average cpm count for each TF. Red color gradient and circle size represent TF expression level. Highly ranked TFs were shown in darker red and bigger circles. **(B)** Genes networks representing transcription factors (TFs) targets, selected from panel A, enriched in the NK RMtsig. TF names are shown in the middle with square red nodes, where each TF (red node) is connected to its targets (gray nodes) with red arrows.

In order to further dissect the NK cell transcriptional programs, we performed an enrichment analysis using the transcription factors (TFs) database pscan (Zambelli et al., 2009). First, we screened for all human TFs and found 332 TFs among NK RMtsig (Supplementary Table 4). Several TFs involved in regulating many biological functions were part of NK RMtsig and include interferon regulatory factors (*IRF7, IRF3, IRF8*), Forkhead transcription factor family (*FOXO1, FOXP1*), several transcriptional activator and repressor (*BHLHE40, TOX, CTCF, SP1, SMAD*), TFs involved in proliferation and activation (AP1, NFAT, JUN, FOS, EOMES) and TFs regulating apoptosis (*FOXO3, HIF1A*). Next, we tested the enrichment of TFs downstream targets in the NK RMtsig using the overlapping test as described above. We found that several transcription factor targets were enriched in our NK RMtsig including TF regulating interferon responses and inflammation, survival, proliferation and activation signaling (Figure 6, Supplementary Table 4). Some of the TFs downstream targets identified in NK RMtsig are illustrated in Figure 6B. Collectively, these data suggest that at a naive state NK cells are poised for rapid responses that are regulated by common TFs and engage in a complex integrative innate immune networks that include metabolism, cytokines and chemokines, TLR and interferon signaling.

### The NHP NK Cell Signature Is Modulated During Acute SIV and ZIKV Infections

**Figure 7:**
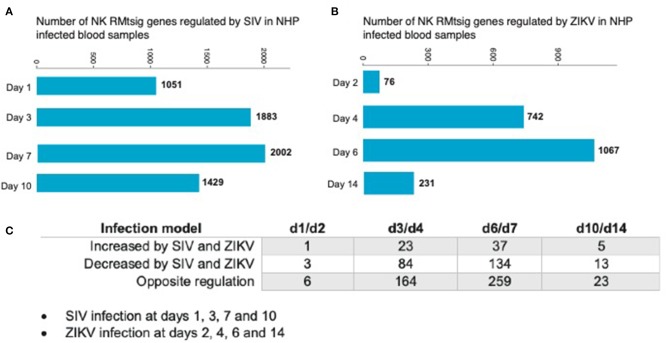
NK RMtsig is modulated following SIV and ZIKV infections in two independent NHP cohorts. **(A,B)** Barplots of the number of NK RMtsig genes that are modulated by SIV **(A)** or ZIKV **(B)** at different time points following infection. **(C)** Number of NK RMtsig genes that are commonly increased or decreased by SIV and ZIKV at different time points following infection.

As an unbiased test case for whether our NK RMtsig could be applied to retrospective longitudinal infection studies, we decided to use the published longitudinal gene expression signatures from two independent NHP infection studies (Barouch et al., 2016; Aid et al., 2017). In the first study (Barouch et al., 2016), microarray experiments were performed on unfractionated mononuclear cells from rhesus macaques intravaginally infected with SIVmac251 taken on days 1, 3, 7, and 10 after infection and at day 0 (before infection) in blood, lymph node (LN) and female reproductive tract (FRT). In the second study (Aid et al., 2017), mononuclear cells were isolated from peripheral blood at days 2, 4, 6, and 14 after intravenous infection with ZIKA virus (ZIKV) and at day 0 (before infection) followed by microarray experiments. Analysis of the first data set revealed that in blood, several NK RMtsig genes were significantly modulated (adjusted *p* < 0.05) following SIV infection at day 1 (1,051 genes), day 3 (1,883 genes), day 7 (2,002 genes) and day 10 (1,429 genes) as compared to day 0 (Figure 7A).

**Figure 8:**
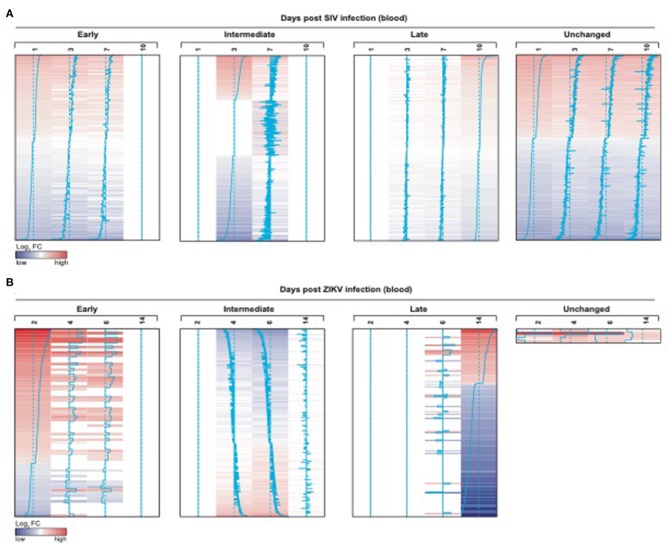
NK RMtsig is modulated following SIV and ZIKV infections in blood from two independent NHP cohorts. **(A,B)** NK RMtsig upregulated (red) or down regulated (blue) genes following SIV infection **(A)** on days 1, 3, 7 and 10 (post-infection) compared to day 0 (before infection) and following ZIKV infection **(B)** on days 2, 4, 6 and 14 (post-infection) compared to day 0 (before infection). Heatmaps represent the log_2_ fold change expression of all significant NK RMtsig genes (Adjusted *p* < 0.05) compared to day 0. Genes were grouped using their expression profiles into 4 clusters: early (day 1 or 2), intermediate (days 3, 4, 6, or 7), late (day 10 or 14) or unchanged.

We also observed significant modulation (adjusted *p* < 0.05) following ZIKV infection at day 2 (76 genes), day 4 (742 genes), day 6 (1067 genes) and day 14 (231 genes) relative to the pre-infection time point, day 0 (Figure 7B). While we observed some similarities in NK RMtsig genes commonly up or down regulated by SIV and ZIKV, several NK RMtsig were regulated in the opposite direction by SIV and ZIKV (Figure 7C). Interestingly, while there were many differences between SIV and ZIKV infection we observed that these up- and down-regulated NK RMtsig genes could be broken down into three phases of modulation: early, intermediate or late responsive genes. The different patterns of modulation ranging from early (day 1 or day 2), intermediate (days 3, 4, 6, or 7), late (day 10 or day 14) or unchanged (similar expression patterns compared to day 0) are shown in Figures 8A,B. In addition, our NK RMtsig showed distinct expression patterns in blood, LN and FRT at different time points following SIV infection suggesting an NK cell tissue specific gene expression profile (Supplementary Figures 4, 5, Supplementary Table 5). Together, these results suggest that our NK cell signature RMtsig could be applied to a wide range of infection models in a highly sensitive manner.

## Discussion

NHP SIV infection models provide an unmatched resource for modeling HIV/AIDS and have been critical to the development of many therapeutic inteventions and vaccine strategies that are currently under development or in use. Because NK cells play a crucial role in regulating viral infections, and the interest in exploiting them for immunotherapeutics, it is important to understand how NK cells respond to viral infections specifically in NHP. Surprisingly, despite the significant work carried out to characterize cellular responses by NK cells, currently there is no substantial unbiased characterization of the NK cell transcriptome in NHP. Prior to this work, NK cell transcriptional profiles have been assessed via RT-PCR or Fluidigm technologies which rely on pre-identified genes of interest (Hong et al., 2013). As such, we set out to first define the identity of an NK cell transcriptomic profile in NHP using RNA-Seq from bulk sorted NK cells to provide an unbiased assessment of the NK cell transcriptome in rhesus macaques. We sorted and performed RNA-Seq on NK cells from experimentally-naïve rhesus macaques in order to minimize animal-to-animal variability. After processing the raw RNA-Seq data, we implemented a statistical method to assign a desirability score for each gene by selecting the most highly expressed genes across all animals in order to minimize any confounding individual-specific responses. This was confirmed via the Spearman rank correlation analysis in Figure 1B, which shows that indeed, there was a low gene expression variability in the NK RMtsig. While it is possible that our selection criteria for determining the genes included in RMtsig may exclude relevant NK cell genes, we decided to use stringent criteria in order to minimize any animal-specific variability and to maximize inclusion of true, NK-specific genes. Nevertheless we provide the full list of genes in the supplemental data along with their desirability scores to serve as a repository for other investigators to assess their genes of interests, if they are not present in RMtsig. We suggest that in some specific setups and experimental designs, where the overall gene expression levels are low, NK genes with low desirabliry scores could also be of interest.

In order to increase confidence that our sorting strategy and mRNA preparation approach was appropriate to characterize the rhesus macaque NK cell transcriptomic signature we decided to compare our signature with several publicly available, NK-specific datasets from human samples (Figures 1C,D, 2). Our analysis revealed that well-established NK cell markers and NK cell signaling pathways were enriched within the NK RMtsig. Through these comparisons we were able to identify genes shared between NK cells in human and NHP, though there are some caveats that must be considered—including differences in cell and RNA preparation, use of different sequencing platforms, and the markers used to sort for NK cells in all these studies. While we did not observe a complete similarity, we were able to identify significant overlap between our NK RMtsig and NK cell signatures identified in the various human studies (Nakaya et al., 2011; Newman et al., 2015; Costanzo et al., 2018; Yang et al., 2019). For instance, in the LM22 study, Newman and colleagues (Newman et al., 2015) defined a molecular signature consisting of 547 genes that distinguish 22 mature human hematopoietic populations isolated from PBMCs from healthy adults including NK cells (CXCR3^+^CD16^+^CD56^+^). While there was substantial overlap between the LM22 NK signature and NK RMtsig, there were 25 genes specific to the LM22 NK signature, including *KIR3DL2, KIR2DL1, KIR2DS4*, and *KLK1*. These markers were either not expressed in our study (cpm=0 or less than the selected significance cut-off) or were highly variable across all animals. The variable gene expression is also partly unsurprising given that the expression of KIR family members has been shown to be extremely variable across individual macaques (Walter and Ansari, 2015). Interestingly, the top overlapping genes between NK RMtsig and all human NK cell signatures include major NK cell markers and signaling networks (Supplementary Table 3), suggesting that our RMtsig is an appropriate signature for rhesus macaque NK cells.

Through this work we identified several signaling mechanisms consistent with NK cell immune responses as evidenced through the enrichment of several cytokines and chemokines, interferon and pathogen sensing pathways (Figures 3, 5). We also observed an enrichment of metabolism, protein secretion, synapse formation and cell survival pathways that support an ability for rapid cellular responses, consistent with perceived NK cell functions (Figure 5). Furthermore, several key transcription factors networks that seem to contribute toward the regulation of many of the identified signaling pathways were enriched in our NK RMtsig (Figure 6). It would be interesting to follow these gene networks over the course of an infection in order to determine whether the presence of viral infections such as HIV or SIV lead to modulation of these key networks, especially during acute infection, where it appears that NK cells are among the earliest immune cells to respond.

Overall in this work we provide the first transcriptomic characterization of NK cells in rhesus macaques, and the first in a non-human primate species. As a result this RMtsig can now be used to interrogate pre-existing datasets even from unfractionated cells. Indeed, we observed that NK RMtsig genes were up or down regulated following intravaginal SIVmac251 infection in blood, LN and FRT tissues and following ZIKV infection in blood in two independent NHP cohorts (Figures 7, 8, Supplementary Figures 4, 5). We anticipate that using RMtsig will provide insight into NK cell responses to disease or future therapeutic interventions through the modulation of the NK RMtsig in unfractionated or NK cell-sorted samples. RMtsig will also serve as a reference point for a “normal” NK cell transcription profile in rhesus macaques. Collectively, this work provides the basis for future NHP infection and disease studies to understand the role of NK cells, and may provide critical insights into informing future human studies of NK cell responses during infection or disease states.

## Data Availability Statement

The datasets generated for this study can be found in the NCBI GEO, accession GSE148290, https://www.ncbi.nlm.nih.gov/geo/query/acc.cgi?acc=GSE148290.

## Ethics Statement

The animal study was reviewed and approved by Harvard Institutional Animal Care and Use Committee and Biomere Institutional Animal Care and Use Committee.

## Author Contributions

RR designed the project. DR processed the PBMCs, sorted NK cells and prepared the RNA for RNA-Seq. SB performed RNA-Seq and generated raw data. MA and DR analyzed the data. MA and DB generated and analyzed whole blood, lymph node and female reproductive tract transcriptomic data. MA, DR, and RR wrote the manuscript.

## Conflict of Interest

The authors declare that the research was conducted in the absence of any commercial or financial relationships that could be construed as a potential conflict of interest.
