# Supplementary material for: Delineation and Modulation of the Natural Killer Cell Transcriptome in Rhesus Macaques During ZIKV and SIV Infections
Source: Front Cell Infect Microbiol. 2020 Apr 29;10:194. doi: 10.3389/fcimb.2020.00194 (PMC7201019; doi:10.3389/fcimb.2020.00194)
Supplement: Supplementary file 1 [file Data_Sheet_1.docx]

**Supplementary Figure 1**

**Gating strategy utilized for identifying and sorting NK cells from naïve rhesus macaque PBMC.** Using a standard approach for rhesus macaques, NK cells were identified and sorted using the following gating strategy: CD14-CD20-CD3-CD159A/C+.

**Supplementary Figure 2**

**RMtsig Desirability plot.** Histogram plot of the log_2_ transformed cpm count of all genes in NK RMtsig. The two (02) red vertical lines represent the minimum cut-off (*cut1= 1*) and the maximum cut-off (*cut2=6*) used to call the *d.high* function and assign a score to each individual gene in RMtsig. All genes with log_2_ (cpm count) < *cut1* were discarded and all genes with log_2_ (cpm count) > *cut2* were assigned a desirability score of 1. Genes with a score between *cut1* and *cut2* were assigned a score using equation 1 as shown in the methods section.

**Supplementary Figure 3**

**Additional pathways enriched the NK RMtsig.** Circular plots showing the enrichment of RMtsig genes in (A) glycosylation and (B) antigen processing and presentation and MHC class I and II pathways.

**Supplementary Figure 4**

**NK RMtsig is modulated following SIV infection in lymph node (LN) and female reproductive tract (FRT) in an independent NHP cohort.** (**A-B**) NK RMtsig upregulated (red) or downregulated (blue) genes following SIV infection in an independent NHP cohort on days 1, 3, 7 and 10 (post-infection) compared to day 0 (before infection) in LN (A) and FRT (B). Heatmaps represent the log_2_ fold change expression of all significant NK RMtsig genes (adjusted p value < 0.05) at days 1, 3, 7 and 10 compared to day 0. Genes were grouped using their expression profiles into 4 clusters: early (day 1), intermediate (days 3-7) , late (day 10) or unchanged.

**Supplementary Figure 5**

**Tissues specific expression of the NK RMtsig following SIV infection on days 1, 3, 7 and 10. (A)** Venn diagrams showing the overlap between the NK RMtsig significantly modulated (increased or decreased) genes in blood, LN and FRT at days 1, 3, 7 and 10 post SIV infection. **(B)** Heatmap of selected NK RMtsig genes increased or decreased on day 10 following SIV infection (adjusted *p* value < 0.05) in blood, LN and FRT.

**Supplementary Figure 6**

**Conserved gene expression between animals.** Correlation matrix showing the pairwise Spearman correlation of the NK RMtsig expression across all six animals. Spearman correlation coefficients are shown for each pair.
